# Effect of Combined Urea and Calcium Nitrate Application on Wheat Tiller Development, Nitrogen Use Efficiency, and Grain Yield

**DOI:** 10.3390/plants14020277

**Published:** 2025-01-18

**Authors:** Chao Wang, Haixing Cui, Min Jin, Jiayu Wang, Chunhui Li, Yongli Luo, Yong Li, Zhenlin Wang

**Affiliations:** 1 Wheat Breeding State Key Laboratory, Shandong Agricultural University, Taian 271000, China; w15066688553@163.com (C.W.);; 2 College of Agronomy, Shandong Agricultural University, Taian 271000, China

**Keywords:** nitrogen (N) form, tiller development, biomass accumulation, hormones

## Abstract

Optimizing nitrogen (N) sources has the potential to improve wheat tillering, nitrogen use efficiency (NUE), and grain yield, yet the underlying mechanisms remain unclear. This study hypothesizes that combining specific N sources can increase zeatin riboside + zeatin (ZR + ZT) content in tiller nodes and maintain a higher ZR + ZT/gibberellin A7 (GA_7_) ratio, thereby promoting tiller development, enhancing NUE, and increasing yield. The effects of N source treatments on two wheat cultivars, the multi-spike Shannong 28 (SN28) and the large-spike Tainong 18 (TN18), were investigated. A total of seven N treatments were tested: no nitrogen (N0), urea (N1), calcium nitrate (N2), ammonium chloride (N3), and equal doses of urea and calcium nitrate (N4), urea and ammonium chloride (N5), and calcium nitrate and ammonium chloride (N6). The results showed that treatment N4 significantly increased the levels of ZR and ZT in tiller nodes, while maintaining a higher ZR + ZT to GA_7_ ratio. This hormonal shift promoted tiller formation and biomass accumulation. Under N4, both cultivars exhibited the highest number of effective spikes and biomass in higher-order tillers. N4 also enhanced N accumulation in the grains, N absorption efficiency, and N translocation, while reducing N loss. Compared to N1, effective spike numbers increased by 7.8% in SN28 and 5.6% in TN18, resulting in a 6.4% increase in grain yield for SN28 and a 2.2% increase for TN18. In conclusion, the combined application of urea and calcium nitrate optimizes hormonal regulation, improves NUE, and significantly enhances wheat tillering and grain yield, providing a promising strategy for enhancing wheat productivity.

## 1. Introduction

Wheat (*Triticum aestivum* L.) is a primary global staple crop, accounting for about 26% of total cereal production worldwide (FAOSTAT, 2022). Nitrogen (N) is a vital macronutrient that significantly influences wheat growth, particularly tillering, a key determinant of final grain yield [1,2,3,4]. Tillers can be classified as effective (able to produce harvestable grains) or ineffective (failing to form ears or grains), highlighting the critical importance of maximizing effective tiller formation [5,6,7].

Different N forms—urea, nitrate (NO_3_^−^-N), and ammonium (NH_4_^+^-N)—vary in their uptake pathways and metabolic effects on plants [8,9]. Different forms of N elicit specific responses in plants. The foliar application of NH_4_^+^-N increases the activity of enzymes and the expression of genes related to N metabolism in wheat flag leaves, whereas the application of NO_3_^−^-N enhances N metabolism and gluten protein synthesis in grains, mobilizing more N from source organs to grains [10,11]. Comparative studies on NH_4_^+^-N and NO_3_^−^-N as primary N sources indicate that NH_4_^+^-N can reduce photosynthesis, respiration, and water demand [12], but it improves plant tolerance to drought stress and pathogens [13,14]. In contrast, NO_3_⁻-N promotes vegetative growth and delays plant senescence. Additionally, compound N fertilizers such as NH_4_^+^-N are more effective in increasing the number of tillers compared to equivalent amounts of single N forms [15]. This suggests that the combination of N forms plays a crucial role in optimizing wheat growth and yield.

The formation of tillers in wheat is regulated by several plant hormones, with cytokinins (CTKs) playing a key role in promoting tiller growth and development [16]. Research has shown that the growth of wheat tiller buds is influenced by the concentrations of auxin (IAA) and zeatin (ZT) at the tiller nodes, as well as the ratios of IAA to ZT and ABA to ZT [17]. Strigolactones (SLs), another critical hormone, regulate plant branching in concentration with IAA and CTK by inhibiting lateral bud growth [18]. In agricultural practice, gibberellin (GA) synthesis inhibitors, such as paclobutrazol, are commonly used to control seedling height and adjust tiller numbers. In rice, gibberellins (GAs) promote stem elongation by facilitating the degradation of SLENDER RICE 1 (SLR1, a key negative regulator of gibberellin signaling) and simultaneously induce the breakdown of MONOCULM1 (MOC1, a key factor promoting tillering), thereby reducing tiller number [19]. Furthermore, GAs can decrease the activity of SNF1-related protein kinases 2 (SnRK2s), which promote the APC/CTE-mediated degradation of SHORT-ROOT1 (OsSHR1, a key factor promoting root growth), ultimately suppressing root growth and tillering [20]. Additionally, GA affects wheat tillering by regulating the biosynthesis of SLs [21]. There is a strong correlation between N forms and endogenous hormones in wheat. Under the same N concentration, NH_4_^+^-N treatment reduced IAA and CTK levels in tobacco roots and shoots compared to NO_3_^−^-N, inhibited cell growth [22], and had a less pronounced effect on zeatin riboside (ZR) increase than NO_3_^−^-N [23]. NO_3_^−^-N influences plant growth and acts as a signaling molecule that stimulates CTK synthesis [24,25]. This indicates that the growth and development of wheat tillers involve complex interactions between different hormones. CTK_S_ and GAs are highly correlated with IAA, abscisic acid (ABA), and SLs. However, it remains unclear whether the changes in the levels and ratios of CTK_S_ and GAs in response to different forms of N during the wheat tillering process affect the growth and development of wheat tillers.

Building upon the critical influence of N sources on wheat hormonal regulation, N uptake, and overall productivity, this study proposes the following hypotheses: (1) The combined application of nitrate nitrogen and urea (N4) can significantly increase ZR + ZT content in tiller nodes and maintain a higher ZR + ZT/GA_7_ ratio, thereby optimizing hormonal balance and promoting wheat tiller development. (2) Treatment N4 enhances N uptake and translocation efficiency, improves N accumulation and utilization efficiency, and simultaneously reduces N loss. (3) The combined application of N4 significantly increases the number of effective spikes and biomass accumulation, ultimately leading to a substantial improvement in wheat yield.

## 2. Results

### 2.1. Pot Experiment Results

#### 2.1.1. Effects of N Sources on the Dynamics of Tiller Growth and Tiller-to-Spike Ratio in Two Wheat Cultivars with Different Spike Types

**Figure 1:**
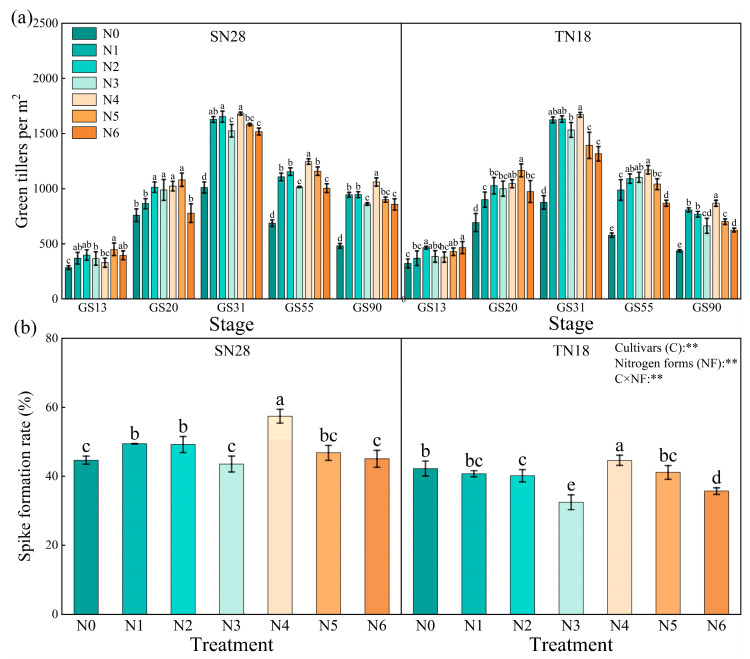
Effects of nitrogen (N) sources on wheat tiller growth dynamics (**a**) and tiller-to-spike ratio (**b**). No nitrogen (N0), urea (N1), calcium nitrate (N2), ammonium chloride (N3), a 1:1 combination of urea and calcium nitrate (N4), a 1:1 combination of urea and ammonium chloride (N5), and a 1:1 combination of calcium nitrate and ammonium chloride (N6). Different letters within a cultivar indicate significant differences at the 0.05 level (*p* < 0.05). C × NF indicates the interaction between cultivars and N forms. The error bar indicates the standard error of the mean (*n* = 3). ** indicate significant differences at *p* < 0.05 and *p* < 0.01.

Both wheat cultivars exhibited a trend characterized by an initial increase followed by a decline in the total number of tillers per unit area across their growth cycles, peaking at the jointing stage (GS31). N application notably stimulated tiller growth at different developmental stages. During the three-leaf stage (GS13), SN28 achieved the highest tiller count with treatment N5, showing a 22.04% and 36.98% increases of over N1 and N4, respectively. TN18’s maximum tiller count was observed with N6, resulting in a 26.06% and 22.71% increase compared to N1 and N4. Both cultivars reached peak tiller counts under N5 during the over-wintering stage (GS20). By GS31, treatment N4 was optimal for enhancing tiller growth in both cultivars. As plants progressed to the heading (GS55) and maturity stage (GS90), treatment N4 yielded significantly higher tiller counts. At GS90, both cultivars consistently responded best to treatment N4. Overall, N5 stimulated the number of tillers during early stages, while N4 facilitated higher emergence rates from GS20 to GS31, benefiting tiller and ear (Figure 1a).

Regarding tiller-to-spike ratio, SN28 exhibited higher levels than TN18 across all treatments. Both cultivars reached peak rates with N4 and the lowest with N3. Despite the differing responses between the two cultivars, treatment N4 significantly enhanced the tiller-to-spike ratios of both cultivars. Compared to other treatments, SN28 showed an increase of 18.37% to 31.82%, while TN18 exhibited an increase of 7.14% to 40.63%, both surpassing the levels observed under other treatments. Consequently, N4 not only increased total tiller counts but also the proportion of effective tillers, contributing to higher yields (Figure 1b).

#### 2.1.2. Effects of N Sources on Dry Matter Accumulation in Tillers at Maturity (GS90)

**Figure 2:**
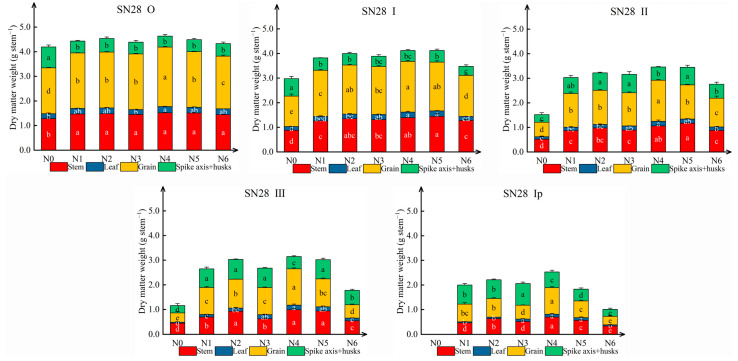
Effects of N sources on the dry matter weight of tillers in different wheat spike types. The data for TN18 are presented in Supplementary Figure S1. No nitrogen (N0), urea (N1), calcium nitrate (N2), ammonium chloride (N3), a 1:1 combination of urea and calcium nitrate (N4), a 1:1 combination of urea and ammonium chloride (N5), and a 1:1 combination of calcium nitrate and ammonium chloride (N6). Wheat plants were separated into main stem (O), first primary tiller (I), second primary tiller (II), third primary tiller (III), and first secondary tiller (Ip). Different letters within a cultivar indicate significant differences at the 0.05 level (*p* < 0.05). The error bar indicates the standard error of the mean (*n* = 3).

Different N forms significantly increased the dry matter accumulation in cultivars’ organs of different tillers in SN28 and TN18 (Figure 2 and Figure S1). As tiller position increased, there was a corresponding decline in dry matter accumulation in all wheat organs. Treatment N4 consistently maximized grain dry weight, ear dry matter, and overall biomass at GS90 for both cultivars, particularly enhancing high-position tillers. Specifically, compared to N1, treatment N4 increased grain dry weight in SN28 by 7.08% to 52.11% and biomass by 4.28% to 27.14% across tillers, with the most pronounced effects on the second primary tiller (II), the third primary tiller (III), and the first secondary tiller (Ip). Similarly, in TN18, N4 outperformed other treatments, elevating grain dry weight by 11.66% to 47.13% and biomass compared to N6, and enhancing ear dry matter accumulation in higher tillers compared to N2. Conversely, treatment N6 yielded the lowest dry matter accumulation and biomass across all tiller organs in both cultivars. While stem and leaf responses to N varied, treatment N4 stood out for significantly boosting biomass, especially in higher-order tillers.

#### 2.1.3. Effects of N Forms on Endogenous Hormones in Tillering Nodes

**Figure 3:**
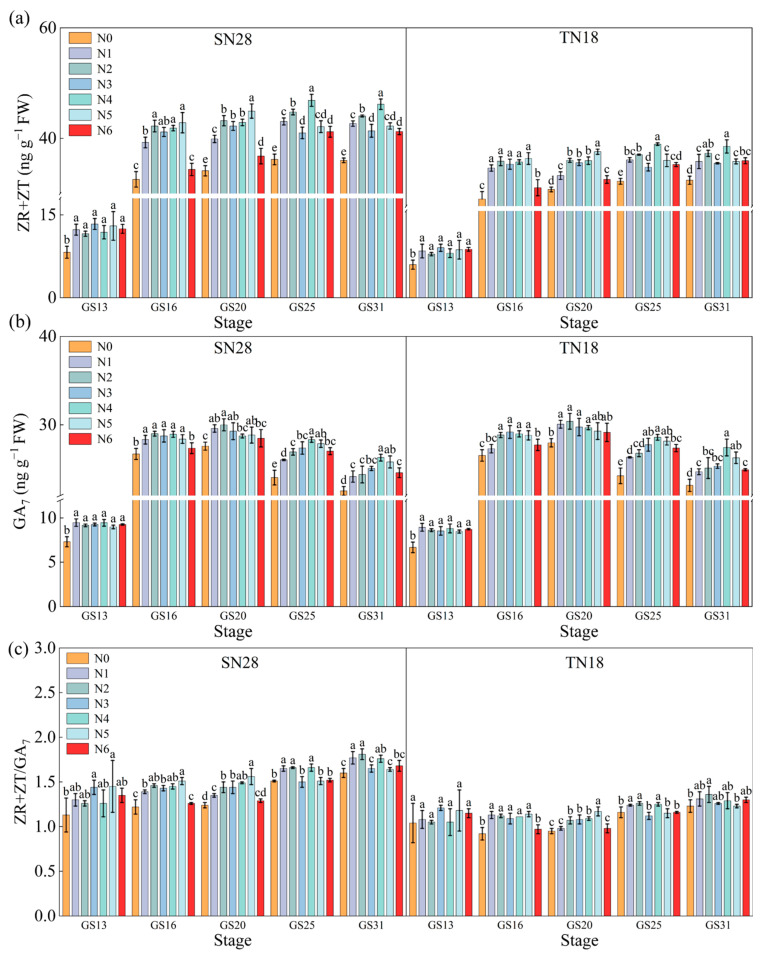
Effect of N sources on the endogenous hormone content in wheat tiller nodes at different growth stages. (**a**) Trans-zeatin-riboside + zeatin (ZR + ZT), (**b**) gibberellin A_7_ (GA_7_), (**c**) ZR + ZT/GA_7_. No nitrogen (N0), urea (N1), calcium nitrate (N2), ammonium chloride (N3), a 1:1 combination of urea and calcium nitrate (N4), a 1:1 combination of urea and ammonium chloride (N5), and a 1:1 combination of calcium nitrate and ammonium chloride (N6). Different letters within a cultivar indicate significant differences at the 0.05 level (*p* < 0.05). The error bar indicates the standard error of the mean (*n* = 3). Tables S1–S3 provides the standard deviation of the data for different treatments, as well as the effects of cultivar, N type, and the interaction between cultivar and N type on the experimental indicators.

From GS13 to the six-leaf stage (GS16) in wheat, the contents of endogenous hormones ZR and ZT in the tillering node rapidly increased, followed by a slower growth in subsequent stages. Compared to N0, N application significantly increased the contents of endogenous ZR and ZT in the tillering node of both wheat cultivars at cultivars growth stages. The contents of endogenous ZR and ZT in the tillering node at GS13 were significantly lower than those at other stages, and there were no significant differences among different N forms. At the GS16, the contents of endogenous ZR and ZT in the tillering node of both cultivars were significantly higher under treatments N2, N3, N4, and N5 than under treatments N1 and N6, but there were no significant differences among the former four treatments. Under treatment N4, the contents of endogenous ZR and ZT in the tillering node of both cultivars were increased by 9.15% and 4.04%, respectively, compared to treatment N1. The response of endogenous ZR and ZT contents in the tillering node of both SN28 and TN18 to N forms at the GS20 stage was consistent with that at the GS16. Treatment N4 significantly increased the contents of endogenous ZR and ZT in the tillering node of SN28 and TN18 at the GS25 and GS31, with increases of 14.80% and 13.60% for SN28, and 11.55% and 14.25% for TN18, compared to treatment N1 (Figure 3a).

The content of endogenous hormone GA_7_ in the tillering node of SN28 and TN18 showed a trend of rapid increase followed by a slow decrease as the growth stage progressed, reaching a maximum during the GS20 stage. N application significantly increased the content of endogenous GA_7_ in the tillering node of both wheat cultivars. At GS13, there were significant differences in endogenous GA_7_ content among the treatments. At the GS16, the contents of endogenous GA_7_ in the tillering node of both cultivars under treatments N1 and N6 were significantly lower than those under other treatments. At the GS20 stage, the content of endogenous GA_7_ in the tillering node of both cultivars under treatment N4 was lower than that under treatments N1, N2, N3, and N5. At the green-up stage, the content of endogenous GA_7_ in the tillering node of SN28 under treatment N4 was significantly higher than that under other treatments, while there were significant differences among the treatments for TN18. At GS31, the contents of endogenous GA_7_ in the tillering node of both cultivars under treatment N4 were significantly higher than those under other treatments, with increases of 8.10% and 11.14% for SN28 and TN18, respectively, compared to treatment N1 (Figure 3b).

From GS13 to GS31, the ratio of (ZT + ZR)/GA_7_ in tiller nodes under treatment N4 for both wheat cultivars remained at a relatively high level, exhibiting a variation trend that was largely consistent with that of ZR + ZT content over the same growth periods (Figure 3c). The ratio of (ZT + ZR)/GA_7_ for SN28 demonstrated an increase of 12.59%, 5.63%, and 4.60% during GS20, GS25, and GS31, respectively, in comparison to treatment N1. Similarly, the (ZT + ZR)/GA_7_ ratio was enhanced by 16.33%, 4.84%, and 2.33% in TN18 during these stages, respectively.

#### 2.1.4. Effects of N Sources on Total N and Total Carbon (C) Content in Different Tillers of Wheat at Maturity (GS90)

##### Total C Content in Different Tillers

**Figure 4:**
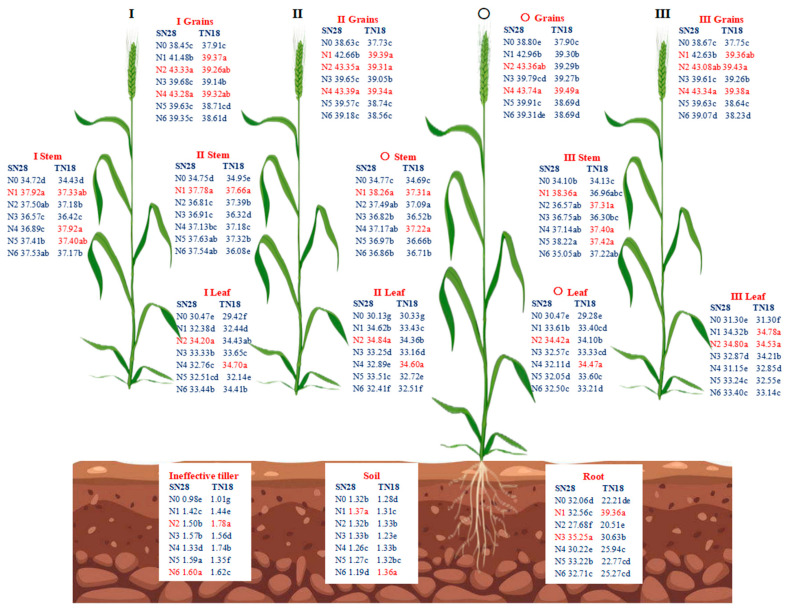
Effects of N sources on total carbon (C, %) of different tillers (the percentage of C content in the measured organ sample mass.). Wheat plants were separated into main stem (O), first primary tiller (I), second primary tiller (II), third primary tiller (III), and first secondary tiller (Ip). No nitrogen (N0), urea (N1), calcium nitrate (N2), ammonium chloride (N3), a 1:1 combination of urea and calcium nitrate (N4), a 1:1 combination of urea and ammonium chloride (N5), and a 1:1 combination of calcium nitrate and ammonium chloride (N6). Different letters within a cultivar indicate significant differences at the 0.05 level (*p* < 0.05). Tables S4–S7 provides the standard deviation of the data for different treatments, as well as the effects of cultivar, N type, and the interaction between cultivar and N type on the experimental indicators. Red markings indicate the treatment with significance level a and the corresponding values.

Compared to N0, N application significantly increased the total C content in the leaves, stems, and grains of the tillers of the two winter wheat cultivars, SN28 and TN18, at GS90 (Figure 4). Under the same N treatment, there were significant differences in the total C content among different tillers of the two cultivars. Specifically, under the same N form treatment, the C content in grains of both SN28 and TN18 was higher than that in stems and leaves. The grain C content of SN28 under treatments N2 and N4 was significantly higher than that under other treatments, but there was a significant difference between the two. The grain C content of tillers I, II, and III of TN18 was relatively higher under treatments N1, N2, and N4, but the differences among treatments were not significant. Additionally, under treatment N4, the total C content in the grains of tiller O was significantly higher than that under other treatments. For the total C content in stems, SN28 reached the maximum value under treatment N1, but the difference compared to other treatments was not significant. The response of stem C content of cultivar tillers of TN18 to N forms showed inconsistency. The total C content in the leaves of SN28 reached the highest value under treatment N2. The total C content in the leaves of tillers O, I, and II of TN18 was the highest under treatment N4, while tiller III reached the maximum value under treatment N2. In terms of root C content, SN28 was significantly higher under treatment N3 than under other treatments. TN18 was significantly higher under treatment N1 than under other treatments.

##### Total N Concentration in Different Tillers

**Figure 5:**
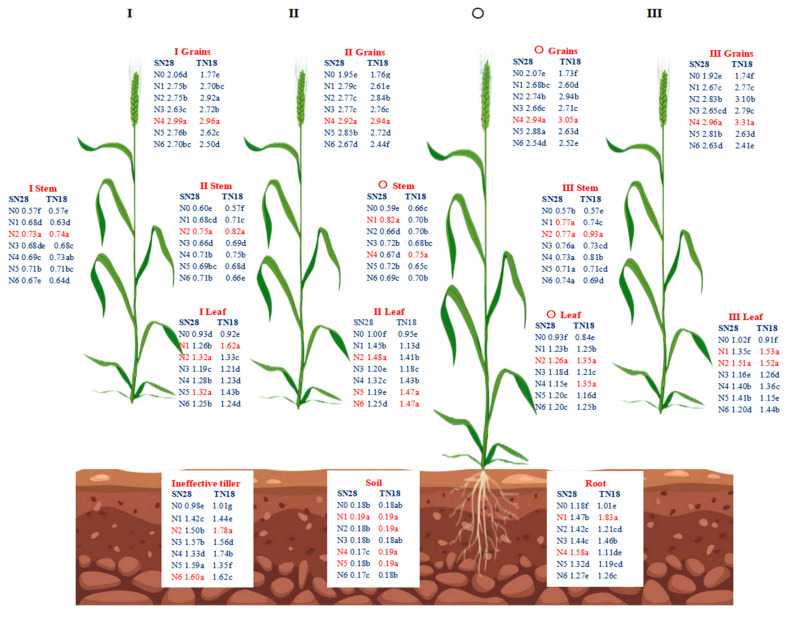
Effects of N sources on total N (%) of different tillers (the percentage of N content in the measured organ sample mass.). Wheat plants were separated into main stem (**O**), first primary tiller (I), second primary tiller (II), third primary tiller (III), and first secondary tiller (Ip). Different letters within a cultivar indicate significant differences at the 0.05 level (*p* < 0.05). Tables S8–S11 provides the standard deviation of the data for different treatments, as well as the effects of cultivar, N type, and the interaction between cultivar and N type on the experimental indicators. Red markings indicate the treatment with significance level a and the corresponding values.

In terms of N concentration, the order of total N concentration in cultivar tiller organs of winter wheat at GS90 under different N treatments was as follows: grain > ineffective tiller > root > leaf > stem > soil (Figure 5). The total N concentration in grains of both SN28 and TN18 was the highest under treatment N4 and the lowest under treatment N0. The response order of total N concentration in the grains of tiller O of SN28 under different N forms was N4 > N5 > N2 > N1 > N3 > N6 > N0. The order of total N concentration in the grains of tillers O, I, and II of TN18 was N4 > N2 > N3 > N5 > N1 > N6 > N0. In terms of total N concentration in leaves, the highest value for tiller O of SN28 was reached under treatment N2. The highest total N concentration in the leaves of tiller O of TN18 was observed under treatment N1. For the total N concentration in stems, the highest value for tiller O of SN28 was observed under treatment N1. The highest total N concentration in the stems of tiller O of TN18 was reached under treatment N4. In terms of total N concentration in roots and soil, the highest N concentration in the roots and ineffective tillers of SN28 was observed under treatments N4 and N6. The highest N concentration in the roots of TN18 was observed under treatment N1.

#### 2.1.5. Effects of N Sources on N Accumulation and Utilization in Wheat

##### N Concentration in Different Organs of Wheat

**Figure 6:**
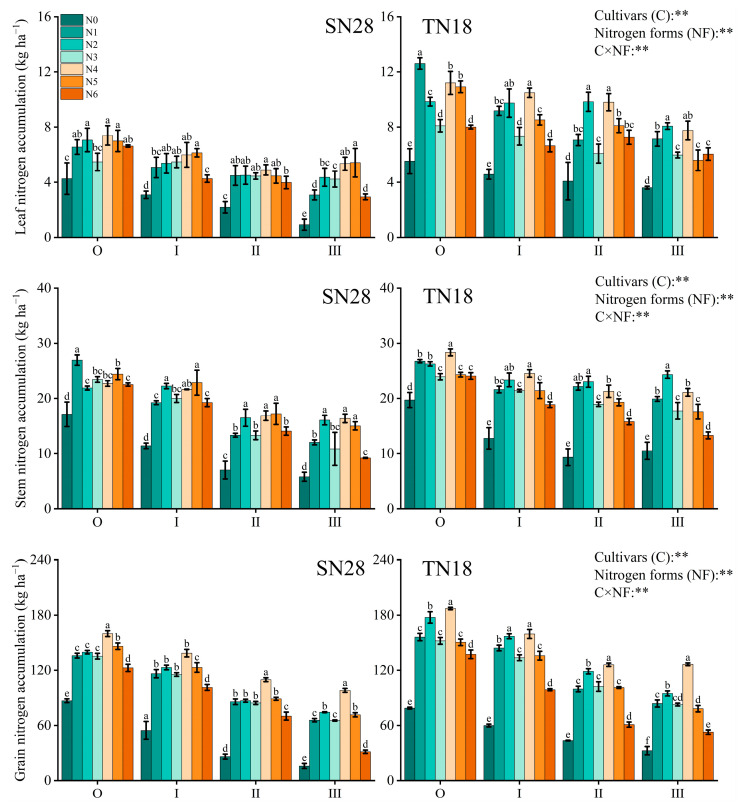
N accumulation in different organs of tillers of wheat at GS90 under different sources of N treatment. Wheat plants were separated into main stem (O), first primary tiller (I), second primary tiller (II), third primary tiller (III), and first secondary tiller (Ip). Different letters within a cultivar indicate significant differences at the 0.05 level (*p* < 0.05). C × NF indicates the interaction between cultivars and N forms. The error bar indicates the standard error of the mean (*n* = 3). ** indicate significant differences at *p* < 0.05 and *p* < 0.01.

The results shown in Figure 4 indicate that, at GS90, N accumulation in various organs of the different tillers for the two wheat cultivars exhibits significant differences in response to the N form. Overall, the N accumulation pattern is as follows: grain > stem > leaf. Compared to N0, all other N treatments significantly increased N accumulation in each tiller organ (Figure 6).

For the large-spike wheat cultivar SN28, treatment N4 significantly increased grain N accumulation, especially in higher-order tillers. The grain N accumulation for tillers O to III increased by 30.42–212.97% and 17.52–49.32%, respectively, compared to treatments N1 and N6. In contrast, the response of N accumulation in leaves and stems to different N forms was more complex. The N accumulation in the leaves of tiller O increased most under treatment N4, with a rise of 73.47% and 35.10% compared to N0 and N3, respectively, and the N accumulation in the leaves of tiller III under treatment N4 was significantly higher than that of other treatments except for N5, with an increase of 38.07–104.76%. The N accumulation in the leaf sheaths was higher under treatments N1 and N4, but differences existed between tiller positions. For instance, tiller III under treatment N4 saw the highest increase at 77.85% compared to other treatments.

For the large-spike winter wheat cultivar TN18, treatment N4 also led to significant increases in grain N accumulation, particularly in high-position tillers. The grain N accumulation for tillers O to III increased by 19.95–288.44% and 36.22–140.29%, respectively, compared to treatments N1 and N6. The response of N accumulation in leaves and stems to different treatments was relatively complex. For example, the leaf N accumulation for tiller O was highest under treatment N1, increasing by 128.44% compared to N0, while for tiller I, leaf N accumulation was highest under treatment N4, with an increase of 129.04%. In terms of stem N accumulation, treatment N4 significantly enhanced levels in low-position tillers, but the increase diminished with higher tiller positions.

In summary, treatment N4 markedly enhanced grain N accumulation in all tillers for both cultivars, particularly in higher-order tillers. The impact on N accumulation in leaves and stems varied depending on tiller position and cultivar, generally showing an advantage in N translocation to grains in higher-order tillers.

##### N Utilization of Wheat

**Figure 7:**
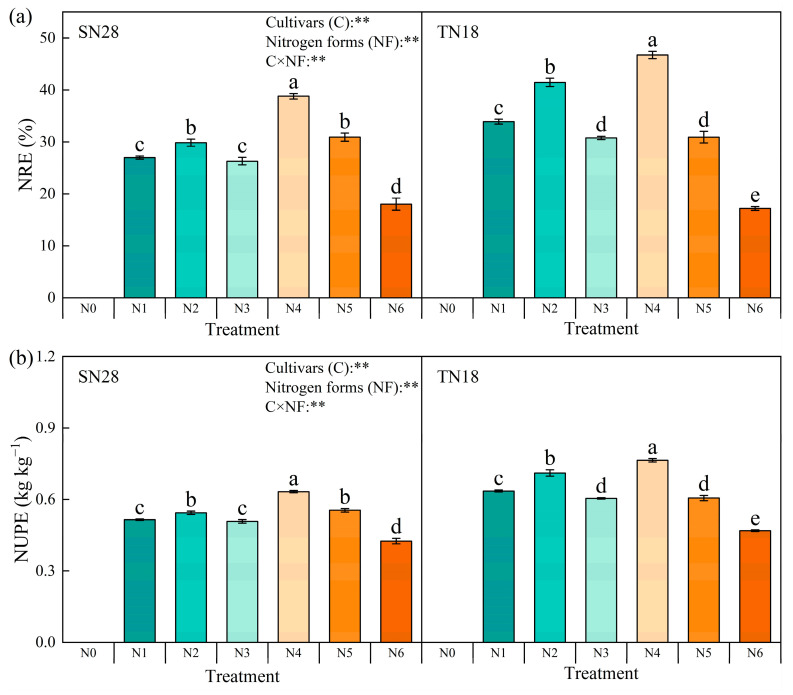
Effects of N sources on nitrogen use efficiency (NUE) of two wheat cultivars with different tillering types. NRE and NUPE represent nitrogen recovery efficiency (**a**) and nitrogen uptake efficiency (**b**), respectively. No nitrogen (N0), urea (N1), calcium nitrate (N2), ammonium chloride (N3), a 1:1 combination of urea and calcium nitrate (N4), a 1:1 combination of urea and ammonium chloride (N5), and a 1:1 combination of calcium nitrate and ammonium chloride (N6). Different letters within a cultivar indicate significant differences at the 0.05 level (*p* < 0.05). C × NF indicates the interaction between cultivars and N forms. The error bar indicates the standard error of the mean (*n* = 3). ** indicate significant differences at *p* < 0.05 and *p* < 0.01.

The effects of different N forms on wheat NUE were evaluated by systematically analyzing two key parameters: nitrogen recovery efficiency (NRE) and nitrogen uptake efficiency (NUPE). The experimental results showed that the large-spike winter wheat cultivar TN18 exhibited significantly higher NRE compared to the multi-spike cultivar SN28 under the same N treatment conditions. Notably, both cultivars achieved their highest NRE under treatment N4. Compared with treatments N1 and N2, treatment N4 increased NRE in SN28 by 44.19% and 30.38%, and in TN18 by 37.76% and 12.74%, respectively (Figure 7).

In terms of NUPE, TN18 consistently outperformed SN28 across all treatment conditions, with both cultivars displaying optimal performance under treatment N4 and the worst under N6. Compared to N4, treatments N1 and N2 significantly reduced NUPE: by 23.53% and 16.67% in SN28, and by 18.75% and 7.04% in TN18, respectively.

### 2.2. Field Experiment Results

#### 2.2.1. Effects of N Sources on the Dry Matter at Different Growth Stages

**Figure 8:**
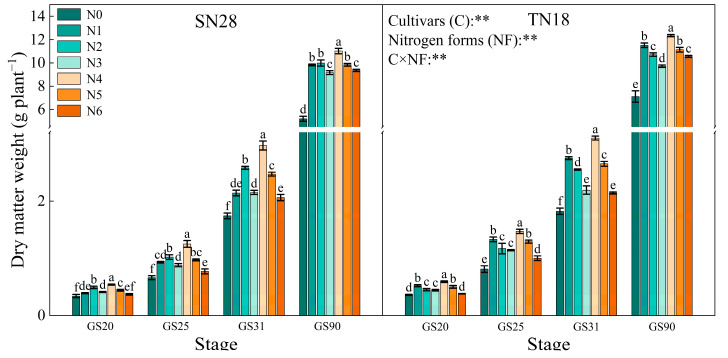
Effects of N sources on dry matter weight of wheat with different spike types. No nitrogen (N0), urea (N1), calcium nitrate (N2), ammonium chloride (N3), a 1:1 combination of urea and calcium nitrate (N4), a 1:1 combination of urea and ammonium chloride (N5), and a 1:1 combination of calcium nitrate and ammonium chloride (N6). Different letters within a cultivar indicate (*p* < 0.05) between treatments in the same stage. C × NF indicates the interaction between cultivars and N forms. The error bar indicates the standard error of the mean (*n* = 3). ** indicate significant differences at *p* < 0.05 and *p* < 0.01.

The dry matter weight of wheat plants continuously increased as they progressed through different growth stages (Figure 8). Across cultivar growth stages and treatments, TN18 exhibited higher plant dry weights than SN28. For both wheat cultivars, the highest plant dry weights were observed under treatment N4 at different growth stages. The plant dry weight of SN28 at different stages followed the order N4 > N2 > N5 > N1, N6, N3 > N0, while for TN18, it was N4 > N1 > N5 > N2, N6, N3 > N0. Compared to N1, the plant dry weight of SN28 and TN18 at GS90 increased by 10.82% and 6.46%, respectively, under treatment N4. Compared to N2, the plant dry weight of SN28 and TN18 at GS90 increased by 9.39% and 12.75%, respectively, under treatment N4.

#### 2.2.2. Yield and Yield Components

Wheat cultivars and N form significantly affected the number of spikes, grains per spike, and thousand-kernel weight, ultimately influencing wheat grain yield (Table 1). Under different treatments, the yield of SN28 was higher than that of TN18. With no N application, wheat spikes, grains per spike, thousand-kernel weight, and yield were the lowest. The highest yields for both wheat cultivars with different spike types were observed under treatment N4. The yield of SN28 followed the order N4 > N1 > N2 > N3 > N6 > N5 > N0, while the yield of TN18 followed the order N4 > N1 > N5 > N6 > N2 > N3 > N0. Under treatment N4, both wheat cultivars had the highest number of spikes, consistent with their yield performance. However, the grains per spike and thousand-grain weight were not consistent with the yield performance, indicating that N4 is beneficial for the formation of effective spikes in wheat, ultimately increasing wheat yield. Compared with the traditional treatment N1, treatment N4 increased the number of wheat spikes by 6.27% (average of both cultivars) and the yield by 4.07% (average of both cultivars).

## 3. Discussion

### 3.1. The N Sources Promote Wheat Tiller Development by Regulating the Ratio of ZR + ZT to GA_7_

The type of N source regulates wheat tiller development by significantly influencing the levels of endogenous hormones in the plant. Specifically, treatment N4 significantly increased ZR + ZT levels in wheat tiller nodes while maintaining a higher ZR + ZT to GA_7_ ratio (Figure 3). ZR + ZT are critical for stimulating meristematic activity in tiller buds by enhancing cell division and elongation in tiller nodes, thereby promoting tiller growth [26,27]. This hormonal regulation significantly promoted tiller development and biomass accumulation.

In contrast, GA_7_ is generally associated with growth inhibition as it suppresses bud growth and reduces resource allocation to tiller buds [28]. However, under treatment N4, the ZR + ZT/GA_7_ ratio in tiller nodes was significantly increased across all growth stages. This hormonal shift optimized the balance between promotive and inhibitory signals, effectively supporting tiller formation while mitigating the suppressive effects of GA_7_ [29]. Additionally, the increase in ZR + ZT levels facilitated more efficient N transport from the roots to the aboveground parts [30], improving N distribution and utilization efficiency (Figure 5, Figure 6 and Figure 7). This shift in hormonal balance enhanced the synergistic effect of tillering development and dry matter accumulation (Figure 2, Figure 4 and Figure 8), thereby improving wheat’s tillering capacity and overall plant growth potential. Compared to other treatments (e.g., N5 and N6), N4 demonstrated a more pronounced effect in increasing ZR + ZT levels and maintaining an optimal ZR + ZT/GA_7_ ratio. This effect may be attributed to the role of nitrate in promoting cytokinin synthesis and the role of urea in providing a sustained N supply to support hormonal regulation [31].

In conclusion, the combined application of urea and calcium nitrate significantly enhanced ZR and ZT levels in tiller nodes, maintained a higher ZR + ZT/GA_7_ ratio, and promoted wheat tiller development. These findings confirm the hypothesis that N4 optimizes hormonal balance and provide a robust theoretical foundation for improving wheat productivity through N management.

### 3.2. Effects of N Sources on N Accumulation and Utilization Efficiency

Treatment N4 significantly increased wheat’s NRE and NUPE (Figure 7), while reducing N loss. This result suggests that N4 optimized wheat’s ability to absorb and utilize N. The effect of N source type on N recovery and utilization efficiency is mainly attributed to the energy efficiency of plant N absorption and assimilation, as well as the dynamic processes of N sources in the soil. Previous studies have shown that NO_3_^−^-N promotes the transport and accumulation of N from roots to shoots, while NH_4_^+^-N, with its lower energy cost for absorption and assimilation, has a higher absorption efficiency [32]. However, the practical application of NH_4_^+^-N may be limited by environmental factors such as ammonia toxicity, which could affect its performance under certain conditions [33]. Treatment N4 improved the efficiency of N transport and accumulation within the plant, enhancing the N absorption capability of tiller organs. It also optimized NRE and NUPE, significantly reducing N loss and waste. The complementary effect of the mixed N sources reduced the negative impacts of a single N source, such as the toxicity of NH_4_^+^-N, thereby maintaining the stability of wheat growth [34]. These functional changes worked together to ultimately promote efficient N utilization in wheat.

In conclusion, the results of this study support its initial hypothesis: treatment N4 can improve N accumulation and utilization efficiency, reduce N loss, and enhance its effective translocation, leading to improved NUE and higher grain yield in wheat.

### 3.3. Effects of N Sources on Wheat Tillering, Dry Matter Accumulation, and Yield

Compared to single N sources, treatment N4 significantly increased wheat tiller number and effective spike number, resulting in a substantial increase in final yield (Table 1). Additionally, this treatment promoted dry matter accumulation (Figure 2 and Figure 8) and exhibited a higher tiller formation rate (Figure 1b). Mixed N sources have a distinct advantage over single N sources in enhancing wheat yield [35,36]. The regulatory effects of different N sources on wheat tillering and yield were notably different. NO_3_^−^-N exhibits higher absorption efficiency and conversion rates, allowing it to be quickly utilized by plants, thereby promoting tiller formation and dry matter accumulation. In contrast, NH_4_^+^-N extends the N supply duration, supporting sustained growth demands [37,38]. Treatment N4 consistently resulted in the highest spike number and grain yield, with yield composition primarily driven by spikes per unit area, in line with previous findings [39]. Treatment N4 promoted an increase in tiller number per unit area, enhanced dry matter accumulation capacity, and significantly increased yield. Treatment N4 optimized the balance of endogenous plant hormones, particularly the ratio of ZR + ZT to GA_7_, significantly promoting tiller development and dry matter accumulation. NO_3_^−^-N was able to elevate CTK levels, thereby stimulating cell division and tiller formation, while NH_4_^+^-N provided a stable N supply that supported continuous growth demands and prevented N loss and volatilization [40]. This optimization of hormonal balance not only facilitated tiller formation but also improved N absorption and translocation efficiency, thereby reducing N loss and further enhancing NUE [41]. Differential responses of wheat cultivars to N sources also reflect cultivar-specific physiological mechanisms. The multi-spike cultivar SN28 achieved yield increases under treatment N4 by increasing the number of spikes per unit area, whereas the large-spike cultivar TN18 primarily achieved yield increases by enhancing the number of spikes per unit area and grains per spike. These results indicate that developing appropriate N management strategies tailored to specific cultivars is key to improving wheat yield and NUE.

In conclusion, the results of this study confirm the hypothesis that, compared to single N sources, treatment N4 improves wheat tillering, dry matter accumulation, and final yield. This strategy optimizes tiller dynamics (Figure 1), regulates hormone balance (Figure 3), and more effectively allocates dry matter (Figure 2 and Figure 8), providing stronger theoretical support for wheat N management. It is important to note that different wheat cultivars exhibit varying responses to N sources. Future research should further evaluate the applicability of mixed N applications and potential management optimization pathways under different physiological and environmental conditions.

### 3.4. Discussion of Limitations

The findings of the field experiments demonstrated that the effects of different N sources on wheat yield and its components varied significantly between the two wheat varieties, with treatment N4 significantly increasing the number of effective spikes and the final yield. The results of the pot trials further validated the field experiment findings, highlighting the regulatory roles of different N forms on tillering dynamics, endogenous hormone levels, N accumulation, and NUE.

Specifically, treatment N4 significantly enhanced the yield of both varieties by increasing the number of spikes per unit area (Table 1). Conversely, the pot trial results demonstrated that different N forms promoted tiller formation and dry matter accumulation by regulating endogenous hormone levels (Figure 2 and Figure 8) during critical growth stages. This finding was further substantiated by the enhanced NUE: treatment N4 exhibited higher NRE and NUPE (Figure 7). This improvement was attributed to the synergistic effect of nitrate and NH_4_^+^-N, which optimized N distribution and coordinated hormone regulation within the plant. Furthermore, the pot trials revealed that ZR + ZT levels increased significantly under treatment N4, maintaining a higher ZR + ZT/GA_7_ ratio. This favorable balance promoted cell division and tiller formation during critical stages of N supply (GS25 and GS31) and was ultimately reflected in increased spike numbers and yield under field conditions. However, it should be noted that pot trials have inherent limitations arising from their specific experimental conditions, which can affect the applicability and interpretation of the results. In pot trials, root growth was restricted by container volume, which likely altered root development and its ability to absorb soil N in terms of root expansion, density, and depth distribution. As a result, the experimental conditions in pot trials diverged from those in natural field environments. Additionally, the absence of natural soil profiles and drainage in pot experiments may have increased the risk of nitrate leaching, possibly resulting in the overestimation or underestimation of NUE under the confined experimental setup.

Under natural field conditions, wheat roots were able to expand freely and dynamically adapt to the spatial distribution of soil N. By contrast, the limited root space in pot trials restricted N uptake pathways and may have led to localized variations in soil N concentrations, which could affect N uptake dynamics and the regulation of endogenous hormone balance. For instance, treatment N4 in pot trials achieved relatively high NRE and NUPE; however, these outcomes may have been influenced by the reduced complexity of N mobility under the confined pot conditions. By comparison, in field conditions, nitrate leaching caused by rainfall or irrigation and volatilization losses of NH_4_^+^-N in heterogeneous soil environments might partially diminish these benefits. Therefore, while pot trials provided important physiological evidence for understanding the regulatory mechanisms of N forms, their applicability to actual agricultural production must be validated using field data.

Furthermore, in pot environments with confined root spaces, root–shoot interaction mechanisms may differ substantially from those observed in field conditions. Previous studies have suggested that limited root development is often associated with increased cell division and higher rates of tiller formation, which could also influence endogenous hormone levels (ZR, ZT, and IAA) [42]. Although significant increases in ZR + ZT levels under treatment N4 were detected in the pot experiments, extrapolating these findings directly to field environments may fail to adequately account for the dynamic root–shoot growth adjustments present under natural conditions. Moreover, excessive nitrate concentrations in the restricted soil volume of pot trials may impose localized competition, potentially altering N distribution and utilization patterns in plants.

To address these limitations, this study combined field and pot trial results to achieve mutual validation. Field trials demonstrated that, even under complex natural conditions, treatment N4 significantly improved yield and its components. Overall, despite the limitations of pot trials, including restricted root space and the potential for nitrate leaching, pot experiments remain indispensable for controlling environmental variables and elucidating the mechanistic pathways of N regulation. The integration of data from both field and pot trials, as undertaken in this study, effectively mitigated the limitations of individual experimental approaches and provides a more comprehensive theoretical basis for optimizing N fertilizer management strategies involving different N forms in wheat.

## 4. Materials and Methods

### 4.1. Plant Growth Conditions

The experiment was conducted at the Agricultural Experimental Farm of Shandong Agricultural University (36°17′ N, 117°17′ E) during 2018 and 2019. The region experiences a temperate monsoon climate, with an average annual temperature of approximately 13 °C and precipitation around 680 mm. The soil is classified as Eutriccambisols [43], containing 97.7 g kg^−1^ of clay, 336.2 g kg^−1^ of sand, and 566.1 g kg^−1^ of silt. Soil samples were taken from 0 to 20 cm depth before seeding for basic soil fertility measurements. The soil contained organic matter of 13.51 g kg^−1^, total nitrogen of 1.23 g kg^−1^, ammonium nitrogen of 6.76 mg kg^−1^, nitrate nitrogen of 13.01 mg kg^−1^, available phosphorus of 9.11 mg kg^−1^, and available potassium of 83.23 mg kg^−1^ with a pH of 7.62. The meteorological information at the experimental site during the trial period is shown in Table 2.

### 4.2. Experimental Design

The experiment was divided into two parts: a field experiment and a pot experiment. Samples were taken at the three-leaf stage (GS13), six-leaf stage (GS16), overwintering stage (GS20), regreening stage (GS25), jointing stage (GS31), heading stage GS55), and maturity stage (GS90), following the stages defined by zadoks et al (1974) [44].

#### 4.2.1. Field Experiment

The field trial adopted a split-plot design with cultivar as the main plot and N forms as the sub-plot. Two wheat cultivars, the multi-spike cultivar Shannong 28 (SN28) and the large-spike cultivar Tainong 18 (TN18), were used as experimental materials (the cultivars’ characteristics are provided in the Supplementary Materials). The experiments were conducted at the agricultural experimental farm of Shandong Agricultural University from October 2018 to June 2019. The seeding was carried out on 12 October 2018, with a planting density of 225 plants per m^−2^. The experimental plots had an area of 9 m^2^ (3 m × 3 m) each, with 10 rows planted per plot with a row spacing of 25 cm. The plots were arranged randomly, and each treatment was replicated three times. Seeds were sown by hand. Seven N fertilizer treatments were set up, namely, no nitrogen (N0), urea (N1), calcium nitrate (N2), ammonium chloride (N3), a 1:1 combination of urea and calcium nitrate (N4), a 1:1 combination of urea and ammonium chloride (N5), and a 1:1 combination of calcium nitrate and ammonium chloride (N6).

The N application rate of 240 kg ha⁻¹ was chosen based on regional agricultural practices and the relevant literature. This rate is commonly used in high-yield wheat fields in the study area to achieve optimal productivity and nitrogen use efficiency [45], applied at a ratio of 1:1 as basal fertilizer and top dressing, with top dressing carried out at the GS31 of wheat. Phosphorus and potassium fertilizers were all applied as basal fertilizers, with a phosphorus (P_2_O_5_) application rate of 100 kg ha^−1^ and a potassium (K_2_O) application rate of 100 kg ha^−1^. Potassium dihydrogen phosphate (P 23%, K 29%) and potassium chloride (containing 60% K_2_O) were used as phosphorus and potassium fertilizers, respectively. Additionally, calcium chloride (CaCl_2_) was applied to each plot to ensure that the same amount of pure calcium was applied to all plots, avoiding experimental errors due to differences in calcium content. Other management practices were the same as those for commercial high-yield fields.

#### 4.2.2. Pot Experiment

The wheat cultivars and N forms used in the pot experiment were the same as those in the field trial. The pots had a diameter of 24 cm and a height of 35 cm. Soil from the cultivated layer of the field was sieved and used, ensuring the same soil volume in each pot. Seeding was carried out on 12 October 2018, with a seeding rate of 15 seeds per pot, and thinning was carried out at GS13 to leave 10 plants per pot. Thirty pots were used for each treatment, and the cultivation methods were the same.

Seven different forms of N, as described above, were used as N sources. The N was applied at a ratio of 1:1 as basal fertilizer and top dressing, with a total N application rate of 240 kg ha^−1^. Phosphorus and potassium fertilizers were applied as basal fertilizers, with the same rates and sources as in the field trial. Additionally, calcium chloride was applied to each pot to ensure that all conditions, except for N form, were the same. The corresponding fertilizers were dissolved in 2 L of water and poured into the pots, which were then covered with an equal amount of soil to minimize N loss. The pots were watered simultaneously, and the water volume was strictly controlled using a measuring cup to ensure that the same amount of water was used each time. Different tillers of potted wheat plants were tagged with labels (to differentiate between individual tillers), with six pots per treatment.

### 4.3. Sample Collection

Field: At the GS20, GS25, GS31, and GS90 stages, 30 plants per treatment were randomly selected for dry matter measurement.

Pot: At the GS13, GS16, GS20, GS25, and GS31 stages, tiller nodes were collected, rapidly frozen in liquid N, and stored at −80 °C for the subsequent determination of endogenous hormone content. Concurrently, distinct wheat tillers were labeled with six pots per treatment (to facilitate the differentiation between individual tillers). At the point of GS90, individual wheat plants were subdivided into the following categories: main stem (O), first primary tiller (I), second primary tiller (II), third primary tiller (III), and first secondary tiller (Ip). Each tiller was then subdivided into four components: the stem, the leaf, the spike axis and husks, and the grain. Measurements were taken for total N, total carbon, and dry matter content.

### 4.4. Determination Parameters and Methods

#### 4.4.1. Soil Properties

Soil organic carbon concentration was measured using the wet oxidation–redox titration method [46], and soil organic matter content was calculated by multiplying the result by 1.724. Soil total N was determined using a continuous flow analyzer (AA3, Seal Analytical, Germany) after digestion with sulfuric acid (H_2_SO_4_) and hydrogen peroxide (H_2_O_2_), while soil nitrate and NH_4_^+^-N were measured using the same analyzer following potassium chloride (KCl) extraction [47]. Available phosphorus was determined using the NaHCO_3_ extraction method (0.5 mol L^−1^), and available potassium was analyzed via ammonium acetate (NH_4_OAc) extraction with flame photometry [48]. Soil pH was measured using a glass electrode in a water–soil suspension with a ratio of 2.5:1 [49].

#### 4.4.2. Wheat Population Dynamics Measurement

At the GS13 stage, three pots of uniformly growing potted wheat plants were selected and marked. The number of tillers was recorded once at the GS16, GS25, GS31, GS55 (50% of plants headed), and GS90 stages.

#### 4.4.3. Aboveground Biomass at Different Growth Stages

At the GS20, GS25, GS31, and GS90 stages, 30 uniformly growing plants were sampled from each treatment in the field. These plants were placed in an oven at 105 °C for 30 min prior to drying to a constant weight at 60 °C before weighing.

#### 4.4.4. Dry Matter Weight of Tiller

At the wheat GS90 stage, 30 marked plants were collected from the pot experiments. The plants were separated based on tiller position and each tiller was further separated into stem, leaf, spike axis + husks, and grain. All plant samples were dried at 105 °C for 30 min, to a constant weight at 60 °C, and then weighed.

#### 4.4.5. Determination of Total N and Total C Contents

At the wheat GS90 stage, 15 marked wheat plants per treatment were collected from the pots. The plants were separated into different tillers, and each tiller was further divided into stem, leaf, grain, root, and ineffective tiller parts. Soil samples were also collected from each pot. All plant samples were dried at 105 °C for 30 min, then dried to a constant weight at 60 °C, ground, sieved, and weighed. Samples about 4 mg were wrapped in small tin capsules for the determination of C and N, using an Elemental Analyzer-Isotope Ratio Mass Spectrometer (EA-IRMS), a stable isotope ratio mass spectrometer (Isoprime100, Isoprime Ltd., UK), and an elemental analyzer (vario MICRO cube, Elementar GmbH, Germany).

#### 4.4.6. Determination of Endogenous Hormones in Tiller Nodes

Determination method for ZR + ZT [50]: Extraction solvent (methanol/water/acetic acid = 80:19:1) and internal standard were added to a centrifuge tube. The mixture was shaken in a constant temperature shaker at 4 °C under dark conditions, followed by centrifugation at 13,000 rpm for 15 min. The supernatant was transferred to a new centrifuge tube, vacuum-dried, and then re-dissolved in pure methanol. The solution was filtered through a 0.22 µm organic membrane filter. The resulting solution was analyzed using a Waters Triple Quadrupole Liquid Chromatography–Mass Spectrometry system (UPLC/XEVO TQ-S, Waters Corporation, USA) with a 5 µL injection.

Determination method for GA_7_ [51]: Extraction solvent (isopropanol/acetic acid = 99:1) and internal standard were added to a centrifuge tube. The mixture was extracted overnight at 4 °C in a constant temperature shaker, followed by centrifugation at 13,200× *g*. The supernatant was transferred to a new centrifuge tube, vacuum-dried, and re-dissolved in pure methanol. The solution was filtered through a 0.22 µm organic membrane filter. The resulting solution was analyzed using a Waters Triple Quadrupole Liquid Chromatography–Mass Spectrometry system (UPLC/XEVO TQ-S, Waters Corporation, USA) with a 5 µL injection.

#### 4.4.7. Wheat Yield and Its Components

Field: At wheat GS90, 30 spikes were randomly selected from each treatment to count the number of grains per spike. From each experimental plot, all plants within a marked 1 m² area were harvested and threshed, to determine grain yield. The number of spikes per unit area was also recorded. After air-drying until the grain moisture content decreased to 12%, the grains were weighed, and the thousand-grain weight was measured.

Pot: At wheat GS90, 30 spikes were randomly selected from each treatment to count the number of grains per spike. For each treatment, all plants from two marked pots were harvested and completely threshed. After air-drying until the grain moisture content decreased to 12%, the grains were weighed, and the thousand-grain weight was measured.

### 4.5. Calculation Formulas for Experimental Parameters

NAO (kg ha^−1^) = NSO × DSO

NRE (%) = (NAA − NAF)/NAR × 100%

NUPE (kg kg^−1^) = NA/NAR

where NAO is the nitrogen accumulation in different tiller organs of wheat plants at GS90 (kg ha^−1^), NSO is the nitrogen content in a tiller organ (kg kg^−1^), DSO is the dry matter weight of that tiller organ (kg ha^−1^), NRE is the nitrogen recovery efficiency (%), NAA is the nitrogen accumulation in wheat plants following nitrogen application (kg ha^−1^), NAF is the nitrogen accumulation in wheat plants in the nitrogen-free area (kg ha^−1^), NUPE is the nitrogen uptake efficiency (kg kg^−1^), NA is the nitrogen accumulation in wheat plants (kg ha^−1^), and NAR is the nitrogen application rate (kg ha^−1^) [52].

### 4.6. Data Statistics and Analysis

A two-way ANOVA was conducted using DPS9.01 Statistical Package (Zhejiang University, Hangzhou, China) on the wheat trait as the response variable and the ‘wheat cultivar’ and ‘N form’ as fixed variables. Multiple comparisons of each indicator under different treatments were performed using the LSD method with a significant probability level of 0.05. The data in figures and tables are the average of three replicates.

## 5. Conclusions

This study highlights the critical role of N forms in regulating tillering, dry matter accumulation, NUE, and yield formation in winter wheat. Specifically, treatment N4 significantly increased the ZR + ZT to GA_7_ ratio, thereby promoting effective tiller formation and enhancing biomass accumulation, particularly in higher-order tillers. Both SN28 and TN18 cultivars exhibited significant improvements in effective spike number. Additionally, treatment N4 accelerated the translocation of dry matter from stems and leaves to grains, ultimately resulting in a substantial increase in grain yield. Furthermore, treatment N4 significantly improved NRE and NUPE, facilitating N accumulation in grains and optimizing nitrogen partitioning. By integrating field and pot experiments, this study effectively bridges the gap between controlled research and practical applications, providing scientific evidence and practical guidance for N management in wheat production. Future research should further explore cultivar-specific responses to N forms, conduct multi-year field trials to validate the stability of the findings, and assess the environmental impacts of optimized N strategies to achieve scalable and sustainable agricultural practices.

## Figures and Tables

**Table 1:** Effect of N sources on yield and its components.

Cultivars	Treatment	Spike Number (m^−2^)	Grain Number per Spike	1000-Grain Weight (g)	Grain Yield (kg ha^−1^)
SN28	N0	600.5 ± 5.62 e	30.89 ± 1.25 c	47.94 ± 0.24 d	7262.33 ± 487.10 d
	N1	760.5 ± 9.71 b	31.79 ± 0.99 c	50.35 ± 0.31 a	9458.30 ± 41.71 ab
	N2	732.5 ± 5.56 bc	33.89 ± 1.02 bc	50.26 ± 0.53 a	9277.50 ± 22.20 b
	N3	665.5 ± 10.78 d	38.86 ± 0.89 a	49.22 ± 0.31 bc	8915.33 ± 92.71 bc
	N4	819.5 ± 3.10 a	36.82 ± 1.19 ab	49.80 ± 0.30 ab	10061.50 ± 149.79 a
	N5	727 ± 12.23 c	32.57 ± 1.29 c	48.48 ± 0.42 cd	8371.37 ± 203.39 c
	N6	717.5 ± 18.03 c	38.93 ± 1.40 a	47.76 ± 0.25 d	8864.23 ± 59.77 bc
TN18	N0	511.5 ± 6.40 f	36.86 ± 1.64 c	41.23 ± 0.35 b	6993.13 ± 149.07 d
	N1	754.5 ± 18.63 b	41.18 ± 1.65 bc	41.51 ± 0.38 b	9157.87 ± 47.55 a
	N2	704.5 ± 3.69 c	44.39 ± 1.86 ab	42.85 ± 0.23 a	8433.23 ± 133.70 b
	N3	575.5 ± 7.41 e	46.79 ± 1.55 ab	41.53 ± 0.25 b	8051.87 ± 59.18 c
	N4	797 ± 6.56 a	47.82 ± 1.51 a	41.91 ± 0.19 b	9357.83 ± 71.92 a
	N5	697.0 ± 7.68 c	45.89 ± 1.66 a	42.87 ± 0.29 a	8714.47 ± 42.14 b
	N6	616.0 ± 14.90 d	45.96 ± 1.21 a	43.36 ± 0.24 a	8682.13 ± 117.89 b
Analysis of variance		*F* values			
Cultivars (C)		8.39 **	1376.72 **	1658.69 **	20.81 **
Nitrogen forms (NF)		14.77 **	107.66 **	7.08 **	48.36 **
C × NF		0.80	14.63 **	11.15 **	3.45 *

Different letters within a cultivar indicate significant differences at the 0.05 level (*p* < 0.05). C × NF indicates the interaction between cultivars and N forms. * and ** indicate significant differences at *p* < 0.05 and *p* < 0.01.

**Table 2:** Meteorological information at the experimental site during the treatment period.

Month	Cumulative Precipitation (mm)	Mean Temperature (°C)	Mean Relative Humidity (%)	Cumulative Sunshine Hours (h)
October 2018	6.7	13.5	61.8	227.3
November 2018	27.9	7.8	77.0	116
December 2018	8.1	0.1	58.8	142.4
January 2019	0.6	−0.9	53.8	151.6
February 2019	2.8	1.3	60.5	113.9
March 2019	3.2	10	44.1	249.2
April 2019	37.1	15	55.8	198.6
May 2019	10.8	21.2	48.6	233.3
June 2019	38.6	27.7	50.1	226.7

## Data Availability

Data are contained within the article and Supplementary Materials.

## Supplementary Materials

The following supporting information can be downloaded at: https://www.mdpi.com/article/10.3390/plants14020277/s1, Figure S1: Effects of N sources on the dry matter weight of tillers in different wheat spike types (TN18); Table S1: Effects of N forms on the endogenous ZR +ZT of wheat with different spike types; Table S2: Effects of N forms on the endogenous GA_7_ of wheat with different spike types; Table S3: Effects of N forms on the ZR+ZT/GA_7_ of wheat with different spike types; Table S4: Effects of N forms on the total leaf carbon of wheat with different spike types; Table S5: Effects of N forms on the total stem carbon of wheat with different spike types; Table S6: Effects of N forms on the total grain carbon of wheat with different spike types; Table S7: Effects of N forms on the ineffective tillers (Ip), roots, soil total carbon of wheat with different spike types; Table S8: Effects of nitrogen forms on the leaf total N of wheat with different spike types; Table S9: Effects of nitrogen forms on the stem total N of wheat with different spike types; Table S10: Effects of N forms on the grain total N of wheat with different spike types; Table S11: Effects of N forms on the ineffective tillers (Ip), roots, soil total N of wheat with different spike types.
