# Supplementary material for: Effect of Combined Urea and Calcium Nitrate Application on Wheat Tiller Development, Nitrogen Use Efficiency, and Grain Yield
Source: Plants (Basel). 2025 Jan 18;14(2):277. doi: 10.3390/plants14020277 (PMC11768312; doi:10.3390/plants14020277)
Supplement: Supplementary file 1 [file plants-14-00277-s001.zip › plants-3415905-supplementary.pdf]

Background of cultivars

Shannong 28

Crop Name: Wheat

Cultivars Name: ShanNong 28

Applicant: Shandong Shengfeng Crop Science Research Institute

Breeders: Shandong Agricultural University, Zibo Hefeng Seed Co., Ltd.

Cultivars Origin: Conventional variety. Developed through systematic selection from a hybridization between Jimai 22 as the female parent and 6125 as the male parent.

Characteristics: Semi-winter type with a total growth period of 226.7 days, maturing around the same time as the control cultivars Zhoumai 18. The seedlings are semi-prostrate with slender leaves, dark green in color, and have strong tillering ability. The plant height is 80.1 cm, with a compact plant type, good lodging resistance, and uniformity. The ears are level, and the maturity appearance is good. The spikes are spindle-shaped with long awns, white grain, hard texture, and plumpness. It has 6.3×10^6 spikes ha^{-1}, 33.4 grains per spike, and a thousand-grain weight of 46.1 g.

Disease Resistance Evaluation: Highly susceptible to sheath blight, highly susceptible to fusarium head blight, highly susceptible to stripe rust, highly susceptible to leaf rust, and moderately susceptible to powdery mildew.

Quality Testing: Grain bulk density of 836 g L^{-1} and 839 g L^{-1} , protein content of 14.58% and 11.79%, wet gluten content of 33.5% and 27.6%, stability

time of 3.0 minutes and 4.0 minutes, and water absorption rate of 64.6% and 60.7%.

Tainong 18

Crop Name: Wheat

Cultivars Name: Tainong 18

Applicants: Taishan District Ruifeng Crop Breeding Research Institute, Tai'an, and College of Agriculture, Shandong Agricultural University

Breeders: Taishan District Ruifeng Crop Breeding Research Institute, Tai'an, and College of Agriculture, Shandong Agricultural University

Cultivars Origin: Conventional variety. Developed through systematic selection from a hybridization between Laizhou 137 as the female parent and Yan 369-7 as the male parent.

Characteristics: Semi-winter type with semi-erect seedlings. Based on the two-year regional trial results, the average growth period is 238 days, maturing 1 day earlier than Weimai 8; plant height is 73.7 cm, with erect leaves, relatively good lodging resistance, and an average maturity appearance. With 4.94×10^6 spikes ha^{-1} effective spikes, and a tiller-to-spike rate of 39.2%. The spike shape is rectangular with 43.6 grains per spike, a thousand-grain weight of 40.8 g, and a bulk density of 795.4 g L^{-1} . It has long awns, white glumes, and white grains that are relatively plump and semi-hard. In 2008, disease resistance evaluation by the Institute of Plant Protection, Chinese Academy of Agricultural Sciences, showed moderate

resistance to fusarium head blight, moderate susceptibility to powdery mildew and sheath blight, and high susceptibility to stripe rust and leaf rust. During the 2007-2008 production trial, samples were uniformly collected and tested by the Ministry of Agriculture Grain Quality Supervision and Testing Center (Tai'an) with results showing a grain protein content of 12.3%, wet gluten of 30.4%, sedimentation value of 33.1 ml, water absorption rate of 59.7 ml $100g^{-1}$, stability time of 6.2 minutes, and flour whiteness of 77.3.

Data from: http://202.127.42.145/bigdataNew/home/ManageOrg.

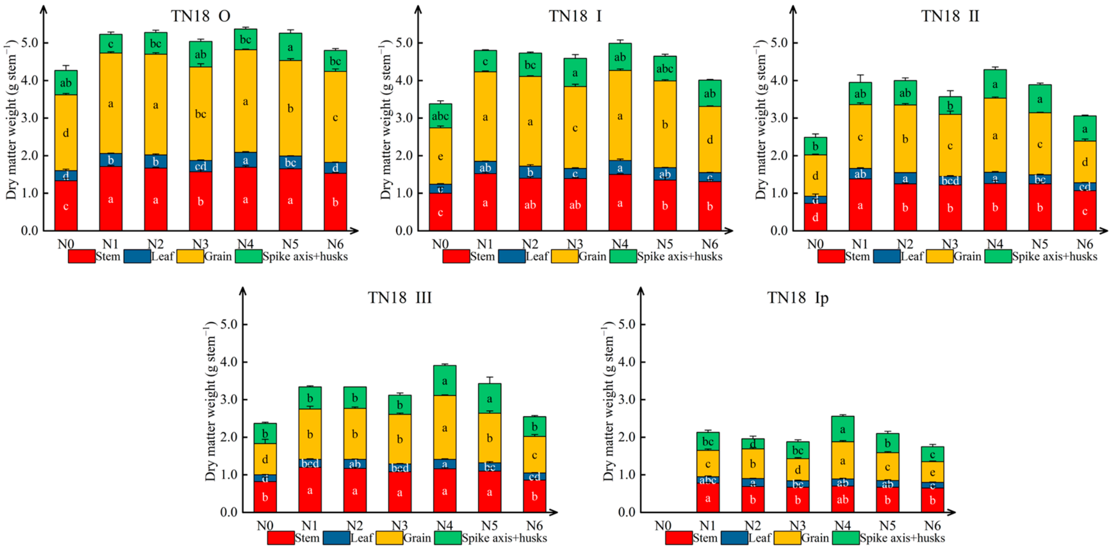

Figure S1. Effects of N sources on the dry matter weight of tillers in different wheat spike types (TN18). No nitrogen (N0), urea (N1), calcium nitrate (N2), ammonium chloride (N3), a 1:1 combination of urea and calcium nitrate (N4), a 1:1 combination of urea and ammonium chloride (N5), and a 1:1 combination of calcium nitrate and ammonium chloride (N6). Wheat plants were separated into main stem (O), first primary tiller (I), second primary tiller (II), third primary tiller (III), and first secondary tiller (Ip). Different letters indicate significant differences at the 0.05 level ($p < 0.05$). The error bar indicates the standard error of the mean ($n = 3$)

Table S1. Effects of N forms on the endogenous ZR +ZT of wheat with different spike types

Cultivars	Treatment	Stage				
		**Three-leaf**	**Tillering**	**Overwintering**	**Turning green**	**Jointing**
**SN28**	N0	8.23 ± 1.08a	32.57 ± 1.40c	34.13 ± 0.93e	36.18 ± 0.97e	36.01 ± 0.42e
	N1	12.32 ± 1.00a	39.25 ± 0.96b	39.88 ± 0.67c	43.05 ± 0.64c	42.67 ± 0.49c
	N2	11.58 ± 0.46a	42.22 ± 1.06a	43.19 ± 0.92b	44.76 ± 0.52b	44.04 ± 0.17b
	N3	13.35 ± 1.00a	41.15 ± 0.82ab	42.25 ± 0.80b	40.99 ± 1.04d	41.36 ± 1.17d
	N4	11.85 ± 1,21a	41.84 ± 0.49a	42.87 ± 0.60b	46.88 ± 1.09a	46.17 ± 0.94a
	N5	13.00 ± 2.60a	42.83 ± 1.84a	44.93 ± 1.29a	42.11 ± 1.05cd	42.24 ± 0.57cd
	N6	12.47 ± 0.81a	34.37 ± 1.11c	36.78 ± 1.40d	41.19 ± 0.99d	41.23 ± 0.56d
**TN18**	N0	6.86 ± 0.95a	24.50 ± 1.54c	26.48 ± 0.49d	28.17 ± 0.61e	28.40 ± 0.84d
	N1	9.68 ± 1.43a	30.92 ± 0.66a	29.34 ± 0.78c	32.63 ± 0.46bc	32.28 ± 1.47c
	N2	9.04 ± 0.35a	32.33 ± 0.93a	32.53 ± 0.41b	33.69 ± 0.11b	33.97 ± 0.65ab
	N3	10.37 ± 0.75a	31.73 ± 1.05a	32.04 ± 0.60b	31.09 ± 0.78d	31.91 ± 0.13c
	N4	9.24 ± 0.91a	32.17 ± 0.46a	32.45 ±0.76b	35.87 ± 0.27a	35.40 ± 1.36a
	N5	9.96 ± 1.93a	32.90 ± 1.21a	34.31 ±0.53a	32.51 ± 1.30c	32.30 ± 0.51c
	N6	10.0 ± 0.39a	26.84 ± 1.67b	28.55 ± 0.79c	31.65 ± 0.42cd	32.43 ± 0.62bc
**Analysis of variance**		***F* values**				
**Cultivars (C)**		42.78**	544.55**	1353.63**	1555.10**	1956.93**
**Nitrogen forms (NF)**		7.95**	60.86**	88.46**	73.78**	84.54**
**C×NF**		0.30	1.09	3.25*	2.49*	3.53*

No nitrogen (N0), urea (N1), calcium nitrate (N2), ammonium chloride (N3), a 1:1 combination of urea and calcium nitrate (N4), a 1:1 combination of urea and ammonium chloride (N5), and a 1:1 combination of calcium nitrate and ammonium chloride (N6). Different letters indicate significant differences at the 0.05 level ($p < 0.05$). C × NF indicate interaction between cultivars and nitrogen forms. The error indicates standard error based on 3 data. * and ** indicate siginificant differences at $p < 0.05$ and $p < 0.01$.

Table S2. Effects of N forms on the endogenous GA_7 of wheat with different spike types

Cultivars	Treatment	Stage				
		Three-leaf	Tillering	Overwintering	Turning green	Jointing
SN28	N0	7.30 ± 0.57b	26.71 ± 0.62b	27.59 ± 0.45c	24.03 ± 0.83e	22.52 ± 0.50d
	N1	9.47 ± 0.42a	28.32 ± 0.49a	29.59 ± 0.42ab	26.03 ± 0.08d	24.15 ± 0.64c
	N2	9.16 ± 0.15a	29.00 ± 0.27a	30.00 ± 0.68a	26.94 ± 0.37c	24.38 ± 0.95c
	N3	9.26 ± 0.16a	28.75 ± 0.69a	29.28 ± 0.95ab	27.38 ± 0.70bc	25.07 ± 0.24bc
	N4	9.45 ± 0.38a	28.93 ± 0.34a	28.74 ± 0.22bc	28.32 ± 0.30a	26.29 ± 0.39a
	N5	8.98 ± 0.21a	28.39 ± 0.49a	28.84 ± 0.91ab	27.87 ± 0.42ab	25.80 ± 0.68ab
	N6	9.26 ± 0.08a	27.36 ± 0.61b	28.48 ± 0.98bc	27.02 ± 0.39bc	24.58 ± 0.55c
TN18	N0	6.67 ± 0.60b	26.54 ± 0.65c	27.97 ± 0.47b	24.23 ± 0.88e	23.15 ± 0.69d
	N1	8.94 ± 0.44a	27.30 ± 0.48bc	30.07 ± 0.44a	26.33 ± 0.08d	24.69 ± 0.33c
	N2	8.62 ± 0.16a	28.85 ± 0.29a	30.40 ± 0.89a	26.79 ± 0.39cd	25.11 ± 1.18bc
	N3	8.54 ± 0.48a	29.19 ± 0.72a	29.75 ± 0.99a	27.75 ± 0.73ab	25.33 ± 0.25bc
	N4	8.81 ± 0.50a	28.98 ± 0.35a	29.68 ± 0.23a	28.59 ± 0.32a	27.44 ± 0.95a
	N5	8.46 ± 0.17a	28.81 ± 0.52a	29.29 ± 0.95ab	28.16 ± 0.44ab	26.28 ± 0.65ab
	N6	8.72 ± 0.08a	27.73 ± 0.64b	29.15 ± 1.03ab	27.37 ± 0.41bc	24.92 ± 0.12c
**Analysis of variance *F* values**						
**Cultivars (C)**		26.80**	0.01	18.38**	2.21	8.84**
**Nitrogen forms (NF)**		25.94**	21.67**	22.16**	48.48**	23.50**
**C × NF**		0.07	1.77	0.36	0.19	0.32

No nitrogen (N0), urea (N1), calcium nitrate (N2), ammonium chloride (N3), a 1:1 combination of urea and calcium nitrate (N4), a 1:1 combination of urea and ammonium chloride (N5), and a 1:1 combination of calcium nitrate and ammonium chloride (N6). Different letters indicate significant differences at the 0.05 level ($p < 0.05$). C × NF indicate interaction between cultivars and nitrogen forms. The error indicates standard error based on 3 data. * and * * indicate siginificant differences at $p < 0.05$ and $p < 0.01$.

Table S3. Effects of N forms on the ZR+ZT/GA_7 of wheat with different spike types

Cultivars	Treatment	Stage				
		**Three-leaf**	**Tillering**	**Overwintering**	**Turning green**	**Jointing**
**SN28**	N0	1.13 ± 0.19b	1.22 ± 0.08c	1.24 ± 0.03d	1.51 ± 0.01b	1.60 ± 0.05c
	N1	1.30 ± 0.07ab	1.39 ± 0.02b	1.35 ± 0.02c	1.65 ± 0.03a	1.77 ± 0.07a
	N2	1.26 ± 0.03a	1.46 ± 0.02ab	1.44 ± 0.06b	1.66 ± 0.01a	1.81 ± 0.06a
	N3	1.44 ± 0.08a	1.43 ± 0.03b	1.44 ± 0.07b	1.50 ± 0.06b	1.65 ± 0.04c
	N4	1.26 ± 0.15ab	1.45 ± 0.03ab	1.49 ± 0.01ab	1.66 ± 0.04a	1.76 ± 0.04ab
	N5	1.45 ± 0.29a	1.51 ± 0.04a	1.56 ± 0.09a	1.51 ± 0.04b	1.64 ± 0.02c
	N6	1.35 ± 0.08ab	1.26 ± 0.01c	1.29 ± 0.02cd	1.52 ± 0.02b	1.68 ± 0.06bc
**TN18**	N0	1.04 ± 0.22a	0.92 ± 0.07b	0.95 ± 0.03c	1.16 ± 0.06b	1.23 ± 0.07b
	N1	1.08 ± 0.10a	1.13 ± 0.04a	0.98 ± 0.02c	1.24 ± 0.01a	1.31 ± 0.08ab
	N2	1.05 ± 0.02a	1.12 ± 0.02a	1.07 ± 0.04b	1.26 ± 0.02a	1.36 ± 0.09a
	N3	1.21 ± 0.03a	1.09 ± 0.06a	1.08 ± 0.05b	1.12 ± 0.04b	1.26 ± 0.01ab
	N4	1.05 ± 0.15a	1.11 ± 0.01a	1.09 ± 0.02b	1.25 ± 0.02a	1.29 ± 0.09ab
	N5	1.18 ± 0.23a	1.14 ± 0.03a	1.17 ± 0.05a	1.15 ± 0.05b	1.23 ± 0.02b
	N6	1.15 ± 0.05a	0.97 ± 0.05b	0.98 ± 0.05c	1.16 ± 0.01b	1.30 ± 0.03ab
**Analysis of variance**		***F* values**				
**Cultivars (C)**		20.1235**	624.9752**	618.1319**	1257.6187**	545.0114**
**Nitrogen forms (NF)**		2.1817	33.3956**	25.6864**	21.8119**	6.7280**
**C × NF**		0.1952	1.3211	1.0703	0.9598	0.7000

No nitrogen (N0), urea (N1), calcium nitrate (N2), ammonium chloride (N3), a 1:1 combination of urea and calcium nitrate (N4), a 1:1 combination of urea and ammonium chloride (N5), and a 1:1 combination of calcium nitrate and ammonium chloride (N6). Different letters indicate significant differences at the 0.05 level ($p < 0.05$). C × NF indicate interaction between cultivars and nitrogen forms. The error indicates standard error based on 3 data. * and * * indicate siginificant differences at $p < 0.05$ and $p < 0.01$.

Table S4. Effects of N forms on the total leaf carbon of wheat with different spike types

Cultivars	Treatment	Tillering			
		O	I	II	III
SN28	N0	30.47 ± 0.41f	30.47 ± 0.11e	29.13 ± 0.21g	31.30 ± 0.17e
	N1	33.61 ± 0.26b	32.38 ± 0.22d	34.62 ± 0.17b	34.32 ± 0.18b
	N2	34.42 ± 0.20a	34.20 ± 0.18a	34.84 ± 0.10a	34.80 ± 0.14a
	N3	32.57 ± 0.34c	33.33 ± 0.09b	33.25 ± 0.01d	32.87 ± 0.09d
	N4	32.11 ± 0.18de	32.76 ± 0.15c	32.89 ± 0.07e	31.15 ± 0.23e
	N5	32.05 ± 0.07e	32.51 ± 0.07d	33.51 ± 0.10c	33.24 ± 0.11c
	N6	32.50 ± 0.06cd	33.44 ± 0.05b	32.41 ± 0.04f	33.40 ± 0.07c
TN18	N0	29.28 ± 0.07e	29.42 ± 0.07f	30.33 ± 0.06g	31.30 ± 0.01g
	N1	33.40 ± 0.11cd	32.44 ± 0.08d	33.43 ± 0.16c	34.78 ± 0.32a
	N2	34.10 ± 0.26b	34.43 ± 0.27ab	34.36 ± 0.16b	34.53 ± 0.04b
	N3	33.33 ± 0.08cd	33.65 ± 0.09c	33.16 ± 0.07d	34.21 ± 0.08c
	N4	34.47 ± 0.04a	34.70 ± 0.10a	34.60 ± 0.12a	32.85 ± 0.09e
	N5	33.60 ± 0.37c	32.14 ± 0.24e	32.72 ± 0.02e	32.55 ± 0.03f
	N6	33.21 ± 0.06d	34.41 ± 0.09b	32.51 ± 0.02f	33.14 ± 0.04d
**Analysis of variance**	***F* values**				
**Cultivars (C)**		73.47**	41.69**	4.78*	1.07
**Nitrogen forms (NF)**		292.71**	596.91**	1557.32**	17.94**
**C×NF**		55.51**	61.24**	169.81**	3.51**

No nitrogen (N0), urea (N1), calcium nitrate (N2), ammonium chloride (N3), a 1:1 combination of urea and calcium nitrate (N4), a 1:1 combination of urea and ammonium chloride (N5), and a 1:1 combination of calcium nitrate and ammonium chloride (N6). Different letters indicate significant differences at the 0.05 level ($p < 0.05$). C × NF indicate interaction between cultivars and nitrogen forms. The error indicates standard error based on 3 data. * and * * indicate siginificant differences at $p < 0.05$ and $p < 0.01$.

Table S5. Effects of N forms on the total stem carbon of wheat with different spike types

Cultivars	Treatment	Tillering			
		O	I	II	III
SN28	N0	34.77 ± 0.14c	34.72 ± 0.19d	34.75 ±0.09d	34.10 ± 0.13b
	N1	38.26 ± 0.36a	37.92 ± 0.20a	37.78 ± 0.37a	38.36 ± 0.13a
	N2	37.49 ± 0.12ab	37.50 ± 0.15b	36.81 ± 0.45c	36.57 ± 0.12ab
	N3	36.82 ± 0.30b	36.57 ± 0.25c	36.91 ± 0.10c	36.75 ± 0.02ab
	N4	37.17 ± 0.40ab	36.89 ± 0.42c	37.13 ± 0.35bc	37.14 ± 0.72ab
	N5	36.97 ± 1.84ab	37.41 ± 0.15b	37.63 ± 0.28ab	38.22 ± 0.06a
	N6	36.86 ± 0.10b	37.53 ± 0.15ab	37.54 ± 0.36ab	35.05 ± 5.27ab
TN18	N0	29.28 ± 0.07e	29.42 ± 0.07f	30.33 ± 0.06g	31.30 ± 0.01g
	N1	33.40 ± 0.11cd	32.44 ± 0.08d	33.43 ± 0.16c	34.78 ± 0.32a
	N2	34.10 ± 0.26b	34.43 ± 0.27ab	34.36 ± 0.16b	34.53 ± 0.04b
	N3	33.33 ± 0.08cd	33.65 ± 0.09c	33.16 ± 0.07d	34.21 ± 0.08c
	N4	34.47 ± 0.04a	34.70 ± 0.10a	34.60 ± 0.12a	32.85 ± 0.09e
	N5	33.60 ± 0.37c	32.14 ± 0.24e	32.72 ± 0.02e	32.55 ± 0.03f
	N6	33.21 ± 0.06d	34.41 ± 0.09b	32.51 ± 0.02f	33.14 ± 0.04d
**Analysis of variance**	***F* values**				
**Cultivars (C)**		1.06	0.22	1.84	0.59
**Nitrogen forms (NF)**		14.34**	54.34**	69.15**	2.29
**C × NF**		1.81	5.95**	20.84**	1.21

No nitrogen (N0), urea (N1), calcium nitrate (N2), ammonium chloride (N3), a 1:1 combination of urea and calcium nitrate (N4), a 1:1 combination of urea and ammonium chloride (N5), and a 1:1 combination of calcium nitrate and ammonium chloride (N6). Different letters indicate significant differences at the 0.05 level ($p < 0.05$). C × NF indicate interaction between cultivars and nitrogen forms. The error indicates standard error based on 3 data. * and * * indicate siginificant differences at $p < 0.05$ and $p < 0.01$.

Table S6. Effects of N forms on the total grain carbon of wheat with different spike types

Cultivars	Treatment	Tillering			
		O	I	II	III
SN28	N0	38.80 ± 0.18e	39.45 ± 0.35c	39.63 ± 0.07c	39.67 ± 0.10c
	N1	42.96 ± 0.05b	41.48 ± 1.66b	42.66 ± 0.98b	42.63 ± 0.63b
	N2	43.36 ± 0.62ab	43.33 ± 0.11a	43.35 ± 0.02a	43.08 ± 0.07ab
	N3	39.79 ± 0.05cd	39.68 ± 0.19c	39.65 ± 0.03c	39.61 ± 0.08c
	N4	43.74 ± 0.46a	43.28 ± 0.43a	43.39 ± 0.14a	43.34 ± 0.12a
	N5	39.91 ± 0.12c	39.63 ± 0.08c	39.57 ± 0.15c	39.63 ± 0.07c
	N6	39.31 ± 0.06de	39.35 ± 0.03c	39.18 ± 0.07c	39.07 ± 0.16d
TN18	N0	38.90 ± 0.03c	38.91 ± 0.06c	38.73 ± 0.16c	38.75 ± 0.04c
	N1	39.30 ± 0.03b	39.37 ± 0.02a	39.39 ± 0.10a	39.36 ± 0.06ab
	N2	39.29 ± 0.16b	39.26 ± 0.11ab	39.31 ± 0.06a	39.43 ± 0.08a
	N3	39.27 ± 0.05b	39.14 ± 0.11b	39.05 ± 0.26b	39.26 ± 0.01b
	N4	39.49 ± 0.18a	39.32 ± 0.25ab	39.34 ± 0.03a	39.38 ± 0.04ab
	N5	38.69 ± 0.07d	38.71 ± 0.09cd	38.74 ± 0.08c	38.64 ± 0.13c
	N6	38.69 ± 0.04d	38.61 ± 0.08d	38.56 ± 0.08c	38.23 ± 0.06d
**Analysis of variance**	***F* values**				
**Cultivars (C)**		848.31**	155.66**	543.80**	1186.03**
**Nitrogen forms (NF)**		168.44**	27.52**	96.31**	223.43**
**C×NF**		103.65**	16.40**	50.80**	102.02**

No nitrogen (N0), urea (N1), calcium nitrate (N2), ammonium chloride (N3), a 1:1 combination of urea and calcium nitrate (N4), a 1:1 combination of urea and ammonium chloride (N5), and a 1:1 combination of calcium nitrate and ammonium chloride (N6). Different letters indicate significant differences at the 0.05 level ($p < 0.05$). C × NF indicate interaction between cultivars and nitrogen forms. The error indicates standard error based on 3 data. * and * * indicate siginificant differences at $p < 0.05$ and $p < 0.01$.

Table S7. Effects of N forms on the ineffective tillers (Ip), roots, soil total carbon of wheat with different spike types

Cultivars	Treatment	IP	Root	Soil
**SN28**	N0	40.42 ± 0.26d	32.06 ± 0.21d	1.32 ± 0.01b
	N1	40.40 ± 0.17d	32.56 ± 0.05c	1.37 ± 0.01a
	N2	41.75 ± 0.15b	27.68 ± 0.03f	1.32 ± 0.03b
	N3	42.23 ± 0.19a	35.25 ± 0.12a	1.33 ± 0.01b
	N4	41.37 ± 0.18c	30.22 ± 0.13e	1.26 ± 0.01c
	N5	41.77 ± 0.13b	33.22 ± 0.21b	1.27 ± 0.01c
	N6	41.61 ± 0.13bc	32.71 ± 0.32c	1.19 ± 0.01d
**TN18**	N0	39.72 ± 0.05e	22.21 ± 0.24de	1.32 ± 0.02d
	N1	41.32 ± 0.18c	39.36 ± 5.30a	1.37 ± 0.01c
	N2	39.89 ± 0.03e	20.51 ± 0.22e	1.32 ± 0.01b
	N3	43.36 ± 0.11a	30.63 ± 0.18b	1.33 ± 0.01e
	N4	40.21 ± 0.15d	25.94 ± 0.43c	1.26 ± 0.01b
	N5	41.59 ± 0.14b	25.36 ± 0.17cd	1.27 ± 0.01bc
	N6	43.13 ± 0.19a	25.27 ± 0.30cd	1.19 ± 0.02a
**Analysis of variance**		***F* values**		
**Cultivars (C)**		0.99	123.87**	20.25**
**Nitrogen forms (NF)**		233.27**	44.19**	22.33**
**C × NF**		98.85**	22.21**	85.04**

No nitrogen (N0), urea (N1), calcium nitrate (N2), ammonium chloride (N3), a 1:1 combination of urea and calcium nitrate (N4), a 1:1 combination of urea and ammonium chloride (N5), and a 1:1 combination of calcium nitrate and ammonium chloride (N6). Different letters indicate significant differences at the 0.05 level ($p < 0.05$). C × NF indicate interaction between cultivars and nitrogen forms. The error indicates standard error based on 3 data. * and * * indicate siginificant differences at $p < 0.05$ and $p < 0.01$.

Table S8. Effects of nitrogen forms on the leaf total N of wheat with different spike types

Cultivars	Treatment	Tillering			
		O	I	II	III
SN28	N0	0.93 ± 0.01d	0.93 ± 0.01f	1.00 ±0.01f	1.02 ± 0.01f
	N1	1.26 ± 0.03b	1.23 ± 0.01b	1.45 ± 0.01b	1.35 ± 0.01c
	N2	1.32 ± 0.04a	1.26 ± 0.01a	1.48 ± 0.01a	1.51 ± 0.02a
	N3	1.19 ± 0.01c	1.18 ± 0.01d	1.20 ± 0.01e	1.16 ± 0.01e
	N4	1.28 ± 0.02b	1.15 ± 0.02e	1.32 ± 0.01c	1.40 ± 0.02b
	N5	1.32 ± 0.01a	1.20 ± 0.02c	1.19 ± 0.01e	1.41 ± 0.01b
	N6	1.25 ± 0.01b	1.20 ± 0.01c	1.25 ± 0.01d	1.20 ± 0.02d
TN18	N0	0.92 ± 0.01d	0.84 ± 0.01e	0.95 ± 0.01e	0.91 ± 0.02f
	N1	1.62 ± 0.01a	1.25 ± 0.02b	1.13 ± 0.01d	1.53 ± 0.01a
	N2	1.33 ± 0.05c	1.35 ± 0.03a	1.41 ± 0.03b	1.52 ± 0.02a
	N3	1.21 ± 0.01d	1.21 ± 0.01c	1.18 ± 0.01c	1.26 ± 0.01d
	N4	1.23 ± 0.02d	1.35 ± 0.01a	1.43 ± 0.02b	1.36 ± 0.01c
	N5	1.43 ± 0.01b	1.16 ± 0.01d	1.47 ± 0.01a	1.15 ± 0.01e
	N6	1.24 ± 0.01d	1.25 ± 0.01b	1.47 ± 0.02a	1.44 ± 0.01b
**Analysis of variance**	***F* values**				
**Cultivars (C)**		98.48**	74.98**	33.16**	24.08**
**Nitrogen forms (NF)**		388.10**	654.22**	1051.17**	1421.15**
**C × NF**		66.16**	74.25**	441.16**	333.70**

No nitrogen (N0), urea (N1), calcium nitrate (N2), ammonium chloride (N3), a 1:1 combination of urea and calcium nitrate (N4), a 1:1 combination of urea and ammonium chloride (N5), and a 1:1 combination of calcium nitrate and ammonium chloride (N6). Different letters indicate significant differences at the 0.05 level ($p < 0.05$). C × NF indicate interaction between cultivars and nitrogen forms. The error indicates standard error based on 3 data. * and * * indicate siginificant differences at $p < 0.05$ and $p < 0.01$.

Table S9. Effects of nitrogen forms on the stem total N of wheat with different spike types

Cultivars	Treatment	Tillering			
		O	I	II	III
SN28	N0	0.59 ± 0.02e	0.57 ± 0.01f	0.60 ± 0.02e	0.57 ± 0.01b
	N1	0.82 ± 0.01a	0.68 ± 0.01d	0.68 ± 0.01cd	0.77 ± 0.03a
	N2	0.66 ± 0.01d	0.73 ± 0.01a	0.75 ± 0.02a	0.77 ± 0.02a
	N3	0.72 ± 0.01b	0.68 ± 0.01de	0.66 ± 0.01d	0.76 ± 0.17a
	N4	0.67 ± 0.02d	0.69 ± 0.01c	0.71 ± 0.01b	0.73 ± 0.01a
	N5	0.72 ± 0.01b	0.71 ± 0.01b	0.69 ± 0.01bc	0.71 ± 0.01a
	N6	0.69 ± 0.01c	0.67 ± 0.01e	0.71 ± 0.02b	0.74 ± 0.01a
TN18	N0	0.66 ± 0.02c	0.57 ± 0.02e	0.57 ± 0.01f	0.57 ± 0.01e
	N1	0.70 ± 0.01b	0.63 ± 0.01d	0.71 ± 0.02c	0.74 ± 0.04c
	N2	0.70 ± 0.01b	0.74 ± 0.03a	0.82 ± 0.02a	0.93 ± 0.02a
	N3	0.68 ± 0.01bc	0.68 ± 0.01c	0.69 ± 0.01d	0.73 ± 0.03c
	N4	0.75 ± 0.03a	0.73 ± 0.02ab	0.75 ± 0.01b	0.81 ± 0.01b
	N5	0.65 ± 0.01c	0.71 ± 0.01bc	0.68 ±0.01d	0.71 ± 0.03cd
	N6	0.70 ± 0.01b	0.64 ± 0.01d	0.66 ± 0.01e	0.69 ± 0.01d
**Analysis of variance**	***F* values**				
**Cultivars (C)**		1.68	1.62	9.62**	1.07
**Nitrogen forms (NF)**		53.38**	100.58**	141.43**	17.94**
**C × NF**		47.87**	5.98**	18.98**	3.51**

No nitrogen (N0), urea (N1), calcium nitrate (N2), ammonium chloride (N3), a 1:1 combination of urea and calcium nitrate (N4), a 1:1 combination of urea and ammonium chloride (N5), and a 1:1 combination of calcium nitrate and ammonium chloride (N6). Different letters indicate significant differences at the 0.05 level ($p < 0.05$). C × NF indicate interaction between cultivars and nitrogen forms. The error indicates standard error based on 3 data. * and * * indicate siginificant differences at $p < 0.05$ and $p < 0.01$.

Table S10. Effects of N forms on the grain total N of wheat with different spike types

Cultivars	Treatment	Tillering			
		O	I	II	III
SN28	N0	2.07 ± 0.03e	2.06 ± 0.03d	1.95 ± 0.04e	1.92 ± 0.01e
	N1	2.68 ± 0.05bc	2.75 ± 0.08b	2.79 ± 0.05c	2.67 ± 0.02c
	N2	2.74 ± 0.01b	2.75 ± 0.02b	2.77 ± 0.02c	2.83 ± 0.01b
	N3	2.66 ± 0.02c	2.63 ± 0.02b	2.77 ± 0.04c	2.65 ± 0.03cd
	N4	2.94 ± 0.02a	2.99 ± 0.06a	2.93 ± 0.02a	2.96 ± 0.02a
	N5	2.88 ± 0.03a	2.76 ± 0.06b	2.85 ± 0.03b	2.81 ± 0.03b
	N6	2.54 ± 0.06d	2.70 ± 0.01bc	2.67 ± 0.03d	2.63 ± 0.01d
TN18	N0	1.73 ± 0.02f	1.77 ± 0.02e	1.76 ± 0.03g	1.74 ± 0.01f
	N1	2.60 ± 0.03d	2.70 ± 0.04b	2.61 ± 0.01e	2.77 ± 0.04c
	N2	2.94 ± 0.06b	2.92 ± 0.07a	2.84 ± 0.04b	3.10 ± 0.03b
	N3	2.71 ± 0.03c	2.72 ± 0.02b	2.76 ± 0.02c	2.79 ± 0.02c
	N4	3.05 ± 0.02a	2.96 ± 0.05a	2.94 ± 0.03a	3.31 ± 0.01a
	N5	2.63 ± 0.01d	2.62 ± 0.06c	2.72 ± 0.01d	2.63 ± 0.02d
	N6	2.52 ± 0.02e	2.50 ± 0.01d	2.44 ± 0.02f	2.41 ± 0.02e
**Analysis of variance**	***F* values**				
**Cultivars (C)**		20.62**	20.44**	146.15**	39.49**
**Nitrogen forms (NF)**		662.27**	324.08**	1245.93**	2500.15**
**C × NF**		50.47**	17.89**	32.98**	195.25**

No nitrogen (N0), urea (N1), calcium nitrate (N2), ammonium chloride (N3), a 1:1 combination of urea and calcium nitrate (N4), a 1:1 combination of urea and ammonium chloride (N5), and a 1:1 combination of calcium nitrate and ammonium chloride (N6). Different letters indicate significant differences at the 0.05 level ($p < 0.05$). C × NF indicate interaction between cultivars and nitrogen forms. The error indicates standard error based on 3 data. * and * * indicate siginificant differences at $p < 0.05$ and $p < 0.01$.

Table S11. Effects of N forms on the ineffective tillers (Ip), roots, soil total N of wheat with different spike types

Cultivars	Treatment	IP	Root	Soil
SN28	N0	0.98 ± 0.03e	1.18 ± 0.01f	0.18 ± 0.01b
	N1	1.42 ± 0.01c	1.47 ± 0.03b	0.19 ± 0.01a
	N2	1.50 ± 0.02b	1.42 ± 0.01c	0.18 ± 0.01b
	N3	1.57 ± 0.01a	1.44 ± 0.01c	0.18 ± 0.01b
	N4	1.33 ± 0.02d	1.58 ± 0.01a	0.17 ± 0.01c
	N5	1.59 ± 0.02a	1.32 ± 0.01d	0.18 ± 0.01b
	N6	1.60 ± 0.02a	1.27 ± 0.01e	0.17 ± 0.01c
TN18	N0	1.01 ± 0.01g	1.01 ± 0.01e	0.18 ± 0.01ab
	N1	1.44 ± 0.01e	1.83 ± 0.21a	0.19 ± 0.01ab
	N2	1.78 ± 0.01a	1.21 ± 0.01cd	0.19 ± 0.01ab
	N3	1.56 ± 0.01d	1.46 ± 0.02b	0.18 ± 0.01ab
	N4	1.74 ± 0.01b	1.11 ± 0.01de	0.19 ± 0.01a
	N5	1.35 ± 0.02f	1.19 ± 0.01cd	0.19 ± 0.01a
	N6	1.62 ± 0.01c	1.26 ± 0.02c	0.18 ± 0.01b
**Analysis of variance**		***F* values**		
**Cultivars (C)**		268.33**	26.11**	52.83**
**Nitrogen forms (NF)**		1355.86**	53.50**	7.43**
**C × NF**		325.98**	28.89**	5.64**

No nitrogen (N0), urea (N1), calcium nitrate (N2), ammonium chloride (N3), a 1:1 combination of urea and calcium nitrate (N4), a 1:1 combination of urea and ammonium chloride (N5), and a 1:1 combination of calcium nitrate and ammonium chloride (N6). Different letters indicate significant differences at the 0.05 level ($p < 0.05$). C × NF indicate interaction between cultivars and nitrogen forms. The error indicates standard error based on 3 data. * and * * indicate siginificant differences at $p < 0.05$ and $p < 0.01$.
